# Cough Sounds Recorded via Smart Devices as Useful Non-Invasive Digital Biomarkers of Aspiration Risk: A Case Report

**DOI:** 10.3390/s21238056

**Published:** 2021-12-02

**Authors:** Hye-Seon Kang, Eung-Gu Lee, Cheol-Ki Kim, Andy Jung, Catherine Song, Sun Im

**Affiliations:** 1 Division of Pulmonary and Critical Care Medicine, Department of Internal Medicine, Bucheon St. Mary’s Hospital, College of Medicine, The Catholic University of Korea, Seoul 14647, Korea; beyer_kr@catholic.ac.kr (H.-S.K.); cydonia01@daum.net (E.-G.L.); 2 Department of Rehabilitation Medicine, Bucheon St. Mary’s Hospital, College of Medicine, The Catholic University of Korea, Seoul 14647, Korea; 2mdfeki@gmail.com; 3 Soundable Health, Inc., San Francisco, CA 94105, USA; andy@soundablehealth.com (A.J.); csong@soundablehealth.com (C.S.)

**Keywords:** aspiration pneumonia, cough, digital technology, automatic cough segmentation, telemedicine, case report

## Abstract

Spirometer measurements can reflect cough strength but might not be routinely available for patients with severe neurological or medical conditions. A digital device that can record and help track abnormal cough sound changes serially in a noninvasive but reliable manner would be beneficial for monitoring such individuals. This report includes two cases of respiratory distress whose cough changes were monitored via assessments performed using recordings made with a digital device. The cough sounds were recorded using an iPad (Apple, Cupertino, CA, USA) through an embedded microphone. Cough sounds were recorded at the bedside, with no additional special equipment. The two patients were able to complete the recordings with no complications. The maximum root mean square values obtained from the cough sounds were significantly reduced when both cases were diagnosed with aspiration pneumonia. In contrast, higher values became apparent when the patients demonstrated a less severe status. Based on an analysis of our two cases, the patients’ cough sounds recorded with a commercial digital device show promise as potential digital biomarkers that may reflect aspiration risk related to attenuated cough force. Serial monitoring aided the decision making to resume oral feeding. Future studies should further explore the clinical utility of this technique.

## 1. Introduction

Globally, pneumonia is the most common cause of infectious mortality, with more than two million adults dying from lower respiratory infections [1]. Previously collected data indicate that 60% of community-acquired pneumonia patients and 87% of hospital-acquired pneumonia patients are diagnosed with aspiration pneumonia [2]. Given the expectation that the incidence will continue to increase with the growth of both the total geriatric population and patients surviving a stroke, it has been proposed that evaluation of cough strength is effective in preventing respiratory complications [3].

With recent advances in digital devices, new sophisticated technologies have been introduced to record and analyze the acoustic properties of cough sounds. These have been validated to be useful in monitoring, diagnosis, and evaluation of response to treatment in patients with airway disorders, such as asthma and chronic obstructive pulmonary disease (COPD), or even in patients with coronavirus disease 2019 (COVID-19) [3,4,5,6,7]. Cough sounds can reflect cough intensity, frequency, or characteristics, with automatic cough frequencies used as endpoints and surrogates for primary endpoints. The European Respiratory Society guidelines introduced cough frequency measurements using cough sounds in 2007 [8] as valid markers of respiratory status.

However, applying these standards to those with a weak cough strength related to hemiparesis can be technically challenging. Contrary to the increased frequency and intensity seen in bronchitis or other respiratory disorders, those with aspiration pneumonia show reduced cough intensity. In particular, patients with swallowing difficulties related to hemiparesis or other neurological disorders can demonstrate inadequate glottic closure [9] and poor respiratory muscle recruitment [10], leading to unsafe airway protection and an inability to generate a proper strong cough to prevent aspirates from entering the airway. Therefore, a different approach is needed to monitor changes in cough intensity in these patients at risk of aspiration pneumonia. A recent review article has shown that weak cough sounds recorded via mobile devices could correlate well with attenuated cough forces as reflected by peak cough flow (PCF) [11]. However, how these cough signs reflect aspiration risk in clinical settings in patients with aspiration pneumonia and how they correlate with clinical changes have not been explored. Those with severe neurological disabilities and increased age and stroke severity are at great risk for aspiration pneumonia. A digital device that can record and help track these changes serially in a noninvasive but reliable manner would be beneficial for monitoring such individuals.

Recent studies have advocated the use of cough sounds to classify those with variable respiratory disorders. We hypothesized that cough sounds recorded via digital devices could help reflect the weak cough sounds related to aspiration pneumonia, and that serial recording of these sounds could reflect either deterioration or improvement of aspiration risk.

## 2. Materials and Methods

### 2.1. Case 1

**Figure 1:**
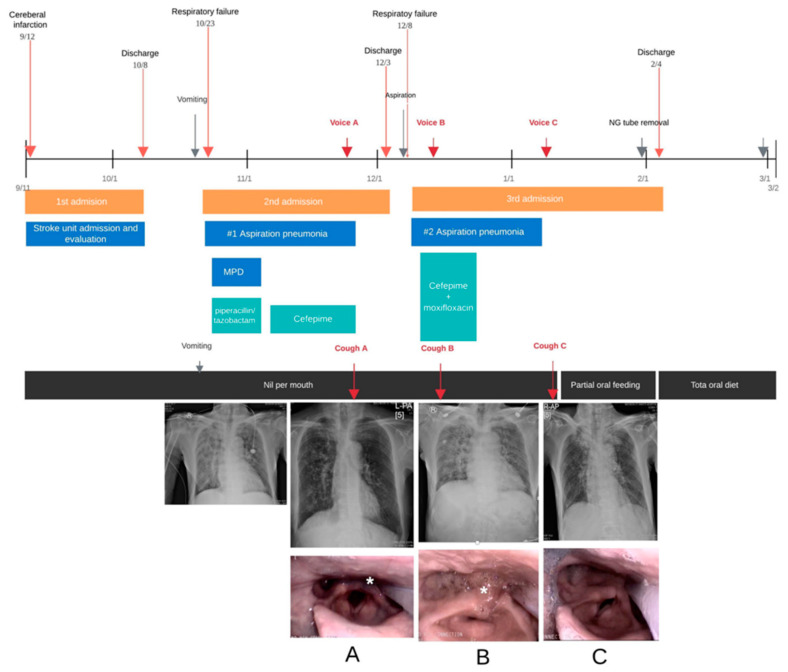
The timeline shows the clinical course of Case 1, with images taken during the FEES examination showing secretion aspiration (asterisk), but with improved swallowing after tube removal. The time points at which cough sounds were recorded are shown, (**A**) before aggravation, (**B**) at aggravation and poor coughing out state and (**C**), at recovery of cough. FEES = fiberoptic endoscopic evaluation of swallowing; MPD = methylprednisolone.

A 76-year-old man was admitted to the Department of Pulmonology due to dyspnea and respiratory distress. Chest radiography revealed infiltration in both lower lung fields, compatible with aspiration pneumonia. The patient was intubated and received antibiotics. He had been on a full feeding tube for five weeks after being diagnosed, with an infarction in the right middle cerebral artery territory. One day before readmission, he had aspirated vomit into his lungs. After two weeks of antibiotic treatment, he showed improvement on chest radiography. A fiberoptic endoscopic evaluation of swallowing (FEES) performed prior to discharge confirmed neurogenic dysphagia and showed aspiration in liquid form; otherwise, he showed tolerable swallowing of puree forms with a functional oral intake scale (FOIS) level of 2 [12], which allowed only minimal bolus training during therapy, and he was mainly dependent on tube feeding. Otherwise, he showed no accumulation of saliva. He was discharged to a rehabilitation facility with instructions for swallowing training. A recording of his cough sound confirmed strong sounds and suggested that he could clear his secretions (Figure 1, cough A).

Two days after discharge, he was readmitted because of cyanosis and dyspnea related to recurrent aspiration pneumonia. The patient had inadvertently ingested large amounts of liquid. Subsequently, a chest radiograph showed increased haziness in the right lung field relative to that on the previous image collected at discharge. Bronchoscopy was performed for toileting, and bacterial culture was conducted to direct targeted antibiotic therapy. At the point of aggravation, he could not clear his secretions, and a cough recording (cough B) demonstrated markedly weaker sounds than those present in the recording obtained days before his discharge.

A follow-up FEES at this time showed increased collection of pulmonary secretion around the arytenoids, which the patient was unable to clear despite attempts. A strict nil per mouth status, with total dependency on tube feeding, was prescribed. The patient started to receive swallowing training along with respiratory physiotherapy to help improve his voluntary coughing.

After 10 days of antibiotic therapy, his chest radiograph revealed improvement. In addition, a follow-up recording of his cough sounds (cough C) included more robust cough sounds. A subsequent FEES examination suggested improvement in both secretion management and swallowing, and he was able to commence oral bolus training. After a few weeks, his swallowing and cough levels improved to a level that allowed nasogastric tube removal and tolerance to an oral diet. Two months after discharge, the patient was fully ambulant, with no significant recurrence of aspiration events.

### 2.2. Case 2

A 37-year-old man was admitted due to a loss of consciousness. His initial serum glucose level was greater than 1500 mg/dL at the time of admission. His serum hemoglobin A1c concentration was 11.2%, and he was diagnosed with hyperglycemic hyperosmolar syndrome with naïve diabetes mellitus. His chest X-ray showed diffuse consolidation in both lower lobes, and he was intubated and admitted to the intensive care unit. A brain magnetic resonance imaging (MRI) scan showed signal changes in both globus palladia, attributable to hyperglycemic encephalopathy.

**Figure 2:**
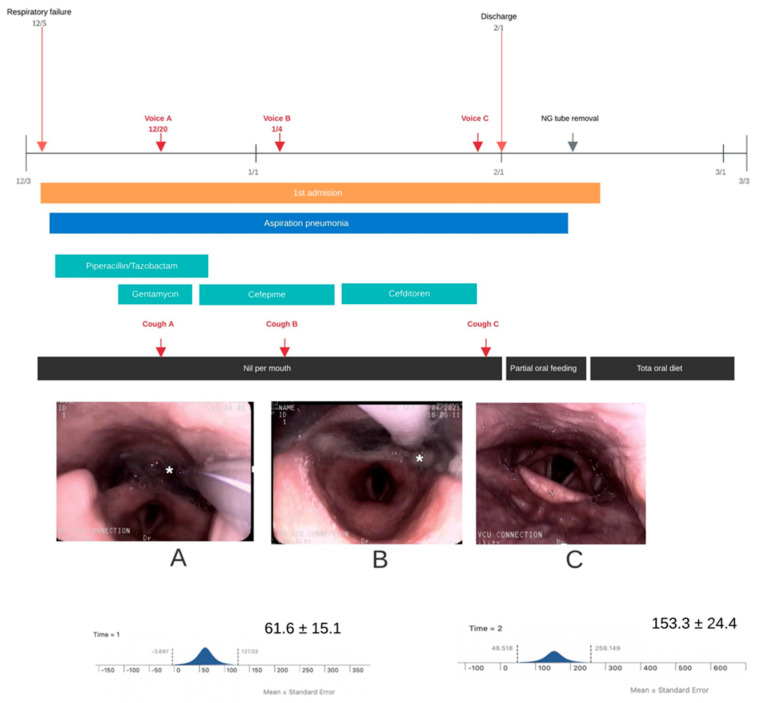
The timeline shows the clinical course of Case 2, with images taken during the FEES examination showing secretion aspiration (asterisk), but with improved swallowing after tube removal and improved peak cough flow. The times at which cough sounds were recorded are shown with cough (**A**) at baseline, (**B**) at follow-up but still with poor cough and cough (**C**) at improvement. FEES = fiberoptic endoscopic evaluation of swallowing; NG = nasogastric.

A nasogastric tube was inserted, and the patient was kept in a nil per mouth status. Days later, he regained consciousness and was referred for a swallowing assessment. At this point, the patient showed profuse secretion and could neither swallow his saliva nor cough out his secretions. A voice recording showed weak cough sounds (Figure 2, cough A), and a FEES examination revealed profuse secretions around the arytenoids without laryngopharyngeal sensation. His laryngeal elevation and pharyngeal contraction showed weakness, with bolus residues remaining in the hypopharyngeal spaces, which indicated silent aspiration past the vocal folds.

A few days later, despite some improvement in his neurological recovery, he showed aspiration of secretions and bolus residues, as manifested by FEES findings and cough sounds (cough B). A spirometric measurement (MicroPlus spirometer; Carefusion Corp., San Diego, CA, USA) at this point indicated a weak mean peak cough flow value of 61.6 ± 15.1 L/min, despite multiple attempts to produce a voluntary cough.

Two days before discharge, a follow-up FEES examination was performed because of frequent self-removal of the nasogastric tube. The findings revealed total resolution of aspiration of secretions and improvement in swallowing. He commenced with a partial oral diet and his swallowing soon recovered to FOIS level 4. Improvement also manifested in cough sounds and a rise in peak cough flow to 153.3 ± 24.4 L/min after multiple attempts at voluntary coughing (cough C).Two months after discharge, he had persistent disabilities in his lower legs related to severe peripheral polyneuropathies but is on a complete oral diet with no significant recurrence of aspiration events. His final FOIS level was 7, and he was able to tolerate a regular diet without any restrictions.

### 2.3. Technical Aspects of Recording

The cough sounds were recorded using an iPad (Apple, Cupertino, CA, USA) with no additional equipment. A voice recorder application provided by Apple was used, and the sampling frequency of the sound was 44,100 Hz. These cough sound signals were band-pass filtered between 20 and 16,000 Hz, and data from the entire frequency band were collected in digital form. Due to the severe medical conditions of our two cases, the cough flow and sound measurements were performed at the bedside with the participants in a semi-reclined position. The distance between the iPad and the patient’s face was kept between 10 and 15 cm. All coughs were recorded in a quiet room to minimize any background sounds.

## 3. Results

**Figure 3:**
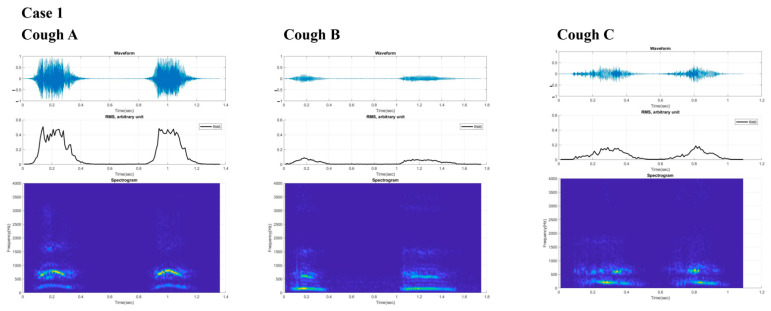
Digital cough sounds from Case 1 are presented for the three times, represented by several values (times) collected on different time points with cough (**A**) before aggravation, (**B**) at aggravation and poor coughing out state and cough (**C**), at recovery of cough. From top to bottom, waveform, root mean square, and spectrogram data of the cough sounds are shown. RMS = root mean square.

**Figure 4:**
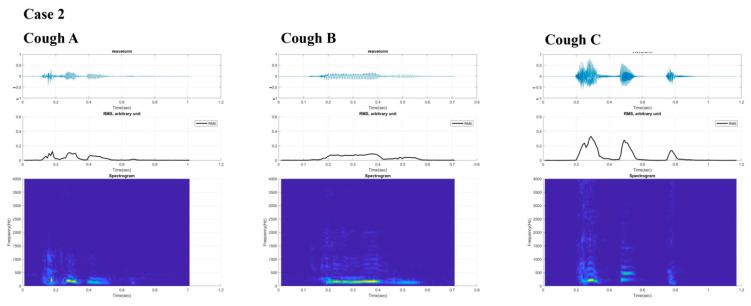
Digital cough sounds from Case 2 are presented for the three times, represented by several values (times) collected on different dates. From top to bottom, waveform, root mean square, and spectrogram data of the cough sounds are shown with cough (**A**) at baseline, (**B**) at follow-up but still with poor cough and cough (**C**) at improvement.

**Figure 5:**
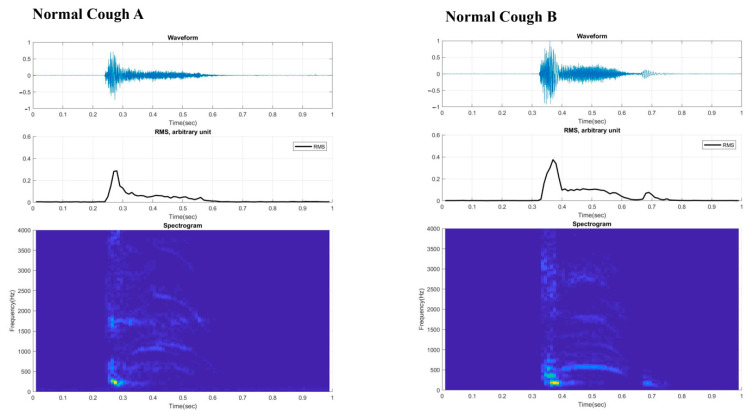
Digital cough sounds from two normal cases for cough (**A**,**B**), that can be divided into two or three phases (explosive, intermediate, and voiced) are shown. RMS = root mean square.

**Figure 6:**
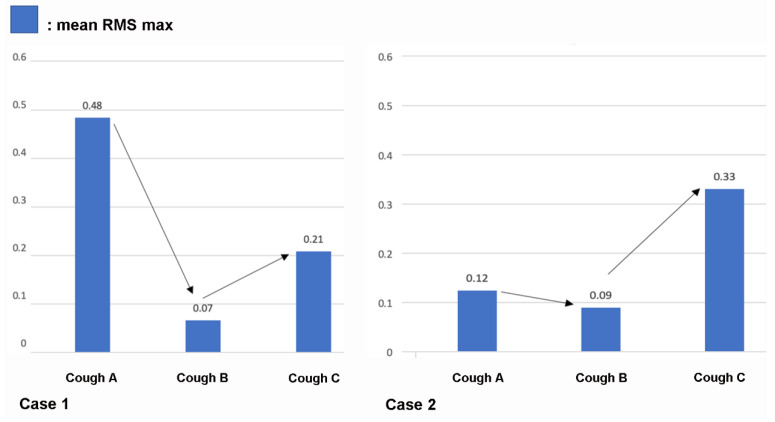
Root mean square (RMS) values presented as a time graph. Maximum RMS values were significantly reduced when the cases were diagnosed with aspiration pneumonia and were higher when the patients’ status improved, and they were able to resume oral feeding.

Figure 3 and Figure 4 present the digital cough sounds represented on a time axis collected on different dates. The waveform, root mean square (RMS), and spectrogram of the cough sounds are shown from top to bottom. According to previous studies, the cough sound waveform can be divided into explosive, intermediate, and voiced phases [8]. In the explosive phase, an abrupt increase in the wave peaks is observed in a typical waveform. However, these separate phases were not observed in the sounds from our two patients. An exception was with the final cough recorded from Case 2 after recovery, which showed some semblance to the those of the normal cases. Typical cough sound waveforms from normal individuals, recorded with the same device (Figure 5), showed robust sound waveforms. When the RMS was represented as a time graph (Figure 6), when noting the acoustic pressure level, the maximum RMS value was reduced when both cases were diagnosed with aspiration pneumonia. In contrast, higher values became apparent with clinical improvement. These figures suggest that maximum RMS values in cough sounds could be helpful to assess the progress and demonstrate a reduced risk of aspiration.

## 4. Discussion

Coughing is the most common symptom of respiratory disease and an important defense mechanism to prevent inhalation or aspiration of foreign material. Moreover, it is critical for both diagnosis and monitoring of the treatment response. Cough frequency can be associated with infection load and is relevant to disease transmission or conversion rates [13]. Changes in cough characteristics can reflect evolving pathological situations and can be used to monitor respiratory status in various pulmonary disorders [13,14]. With the recent advancements in the automatic detection of cough, increased amplitude, frequency, duration, severity, and pattern of cough are relevant features for the automatic detection of respiratory diseases [15]. These techniques can be challenging for those with weak cough sounds and reduced cough frequency related to neurogenic causes. In this case report, we show how serial changes in cough sounds recorded with a digital device, and their RMS values, can be useful in reflecting changes in attenuated cough intensity and characteristics in patients with respiratory failure related to aspiration pneumonia.

While increased coughing and severity indicate progression of respiratory disorders in those with a swallowing impairment, coughing is the primary mechanism protecting the respiratory status from aspiration of foreign bodies, secretions, or food [16]. The cough force, as measured by spirometry findings, has been suggested to correlate well with aspiration risk. Cut-off values below 79 L/min predict respiratory complications with high accuracy [17]. In addition, cough strength, assessed by PCF, can be used to determine the aspiration risk when restarting food intake in elderly subjects with aspiration pneumonia [18]. In our second case, while the PCF level was below the suggested cut-off value in the initial assessment, the value increased in parallel with the mean peak RMS values by the time the patient was allowed to commence oral feeding.

The acoustic properties of cough sounds can reflect cough intensity. In theory, reduced cough sounds can reflect an inability to clear secretions and a poor cough intensity. However, perception levels are still poor [19], and cough sounds are used as an ancillary marker of aspiration risk [20]. Our two cases show that unique cough characteristics, as expressed in the poor distinction of the typical three phases in the cough wave forms, may be of diagnostic value that differentiate coughs associated with aspiration risk from normal typical coughs. Along with the reduced RMS values, one may postulate that these distinctively different cough phases could be essential biomarkers that may help classify aspiration risk and help track the changes in weak cough characteristics across time. In addition, as shown in Case 2, the recovery of the distinctive cough phases from the cough wave forms could indicate improved cough intensity and thus reduced aspiration risk related to poor expectoration.

FEES is a more reliable assessment tool for aspiration and swallowing function; it is a standardized swallowing assessment tool that can also be used to assess the patient’s ability to clear secretions and to determine the risk of intubation [21]. However, it cannot be tested via remote online settings. A similar pitfall can be found in the spirometry measurements used to determine the cough force, which require cooperative patients, experienced staff, and repeated testing to ensure good consistency [22]. In our case reports, only Case 2 was able to undergo PCF measurements, while Case 1 was too fragile to participate.

One major advantage of recording cough sounds using a digital device is the ease of accessibility, with no special equipment or techniques required [23]. Acoustic sounds have been used to detect laryngeal disorders [24] and respiratory disorders [25]. Whereas past studies on automatic detection of cough sounds required the use of heavy equipment, recent advancements in digital devices and the rise in telehealth demand with the COVID-19 pandemic [4,6,7] have facilitated the capabilities of mobile devices to record cough sounds successfully. New techniques can extract features that can be applied to algorithms for diagnosis [5], or community-acquired pneumonia [1]. Our two cases are the first to rely on digital devices to record acoustic cough sounds in aspiration pneumonia. The advantages of requiring minimal patient cooperation and no physical contact make it an ideal approach for patients with significant neurological and physical disabilities.

The recording of cough sounds across serial intervals, as in our two cases, can sufficiently reflect either deterioration or improvement of aspiration risk. In dysphagia, aspiration risk may further be increased by clinical factors such as vomiting, reduced levels of consciousness, old age, and sarcopenia, or even by the level of expertise of staff members [26]. Because these risks might be subject to change depending on improvements in patient neurological status, serial monitoring across various time intervals, rather than a single cough sound assessment, can better reflect changes in swallowing and aspiration risks. The changes in RMS values showed some correlation with the respiratory status of our patients, with low values present during the pneumonia state and higher values recorded after improvements in swallowing and cough. Since there are no digital biomarkers that can reflect aspiration risk, whether serial monitoring of these cough sounds could be used as a digital biomarker to assess and reflect current aspiration status and treatment response in patients is an essential issue that needs to be addressed.

The merits of digital recording of cough sounds in those at risk of aspiration need to be verified by analyzing a larger number of cases. All voice recordings in this study were collected under controlled settings with minimal background noise. Therefore, whether such an approach can be used for remote monitoring with different background noise levels in a heterogeneous environment needs to be assessed. In addition, the correlation of this approach with radiological findings was beyond the scope of this two-case series but is an essential issue that needs to be discussed in future studies. Finally, diagnosing aspiration risk solely based on cough sounds can be challenging, and clinical information should be incorporated in future studies.

## 5. Conclusions

Our two cases demonstrate the unrecognized potential of cough sound-based recognition of those with attenuated cough who are at risk of aspiration pneumonia.

Cough sound recordings of patients with aspiration risk via digital devices are simple to perform and can be applied to remote patient monitoring in telemedicine.

## Data Availability

The datasets used and/or analyzed during the current study have been kept confidential and are not publicly available because the Catholic University of Korea Medical Center does not allow researchers to provide data personally or share them publicly. However, they are available from the corresponding author upon request.

## References

[1] Porter P., Brisbane J.M., Abeyratne U., Wood J., Peltonen V., Bear N., Smith C., Della P., Claxton S. (2020). Diagnosing Community-Acquired Pneumonia: Diagnostic accuracy study of a cough-centred algorithm for use in primary and acute-care consultations. Br. J. Gen. Pract..

[2] Teramoto S., Fukuchi Y., Sasaki H., Sato K., Sekizawa K., Matsuse T., Japanese Study Group on Aspiration Pulmonary D. (2008). High incidence of aspiration pneumonia in community- and hospital-acquired pneumonia in hospitalized patients: A multicenter, prospective study in Japan. J. Am. Geriatr. Soc..

[3] Umayahara Y., Soh Z., Sekikawa K., Kawae T., Otsuka A., Tsuji T. (2020). Clinical Significance of Cough Peak Flow and Its Non-Contact Measurement via Cough Sounds: A Narrative Review. Appl. Sci..

[4] Laguarta J., Hueto F., Subirana B. (2020). COVID-19 Artificial Intelligence Diagnosis Using Only Cough Recordings. IEEE Open J. Eng. Med. Biol..

[5] Rudraraju G., Palreddy S., Mamidgi B., Sripada N.R., Sai Y.P., Vodnala N.K., Haranath S.P. (2020). Cough sound analysis and objective correlation with spirometry and clinical diagnosis. Inform. Med. Unlocked.

[6] Mouawad P., Dubnov T., Dubnov S. (2021). Robust Detection of COVID-19 in Cough Sounds: Using Recurrence Dynamics and Variable Markov Model. SN Comput. Sci..

[7] Rohmetra H., Raghunath N., Narang P., Chamola V., Guizani M., Lakkaniga N.R. (2021). AI-enabled remote monitoring of vital signs for COVID-19: Methods, prospects and challenges. Computing.

[8] Morice A.H., Fontana G.A., Belvisi M.G., Birring S.S., Chung K.F., Dicpinigaitis P.V., Kastelik J.A., McGarvey L.P., Smith J.A., Tatar M. (2007). ERS guidelines on the assessment of cough. Eur. Respir. J..

[9] Han Y.J., Jang Y.J., Park G.Y., Joo Y.H., Im S. (2020). Role of injection laryngoplasty in preventing post-stroke aspiration pneumonia, case series report. Medicine.

[10] Park G.Y., Kim S.R., Kim Y.W., Jo K.W., Lee E.J., Kim Y.M., Im S. (2015). Decreased diaphragm excursion in stroke patients with dysphagia as assessed by M-mode sonography. Arch. Phys. Med. Rehabil..

[11] Umayahara Y., Soh Z., Sekikawa K., Kawae T., Otsuka A., Tsuji T. (2018). A Mobile Cough Strength Evaluation Device Using Cough Sounds. Sensors.

[12] Crary M.A., Mann G.D., Groher M.E. (2005). Initial psychometric assessment of a functional oral intake scale for dysphagia in stroke patients. Arch. Phys. Med. Rehabil..

[13] Lee G.O., Comina G., Hernandez-Cordova G., Naik N., Gayoso O., Ticona E., Coronel J., Evans C.A., Zimic M., Paz-Soldan V.A. (2020). Cough dynamics in adults receiving tuberculosis treatment. PLoS ONE.

[14] Jha R.S., Singh V.P., Mittal V.K. (2017). Discriminant Feature Vectors for Characterizing Ailment Cough vs. Simulated Cough. Proceedings of the 2016 IEEE Region 10 Conference (TENCON).

[15] Shi Y., Liu H., Wang Y., Cai M., Xu W. (2018). Theory and Application of Audio-Based Assessment of Cough. J. Sens..

[16] Bianchi C., Baiardi P., Khirani S., Cantarella G. (2012). Cough peak flow as a predictor of pulmonary morbidity in patients with dysphagia. Am. J. Phys. Med. Rehabil..

[17] Sohn D., Park G.Y., Koo H., Jang Y., Han Y., Im S. (2018). Determining Peak Cough Flow Cutoff Values to Predict Aspiration Pneumonia Among Patients with Dysphagia Using the Citric Acid Reflexive Cough Test. Arch. Phys. Med. Rehabil..

[18] Sakai Y., Ohira M., Yokokawa Y. (2020). Cough Strength Is an Indicator of Aspiration Risk When Restarting Food Intake in Elderly Subjects with Community-Acquired Pneumonia. Respir. Care.

[19] Laciuga H., Brandimore A.E., Troche M.S., Hegland K.W. (2016). Analysis of Clinicians’ Perceptual Cough Evaluation. Dysphagia.

[20] Warnecke T., Im S., Kaiser C., Hamacher C., Oelenberg S., Dziewas R. (2017). Aspiration and dysphagia screening in acute stroke—The Gugging Swallowing Screen revisited. Eur. J. Neurol..

[21] Warnecke T., Dziewas R., Oelenberg S., Ritter M., Dittrich R., Schabitz W.R., Ringelstein E.B., Nabavi D.G. (2006). Serial fiberoptic endoscopic evaluation of swallowing in patients with acute stroke and dysphagia: Case report and general considerations. J. Stroke Cerebrovasc. Dis..

[22] Sharan R.V., Abeyratne U.R., Swarnkar V.R., Claxton S., Hukins C., Porter P. (2018). Predicting spirometry readings using cough sound features and regression. Physiol. Meas..

[23] Sterling M., Rhee H., Bocko M. (2014). Automated Cough Assessment on a Mobile Platform. J. Med. Eng..

[24] Kim H., Jeon J., Han Y.J., Joo Y., Lee J., Lee S., Im S. (2020). Convolutional Neural Network Classifies Pathological Voice Change in Laryngeal Cancer with High Accuracy. J. Clin. Med..

[25] Abeyratne U.R., Swarnkar V., Setyati A., Triasih R. (2013). Cough sound analysis can rapidly diagnose childhood pneumonia. Ann. Biomed. Eng..

[26] Metheny N.A. (2002). Risk Factors for Aspiration. J. Parent. Enter. Nutr..

